# Evidence for the Age-Related Positivity Bias in Autobiographical Memories of the 2020 U.S. Presidential Election

**DOI:** 10.1093/geroni/igaf122.2293

**Published:** 2025-12-31

**Authors:** Karen Siedlecki, Francesca Falzarano, Neshat Yazdani, Jillian Minahan Zucchetto

**Affiliations:** Fordham University, New York, New York, United States; University of Southern California, Los Angeles, California, United States; College Access: Research & Action, New York, New York, United States; Fordham University, New York, New York, United States

## Abstract

The age-related positivity bias refers to the finding that older adults recount events more positively (or less negatively) as compared to younger adults (i.e., a main effect of age on memory valence). This bias is closely related to the positivity effect, which reflects an interaction between age and valence of information to be remembered. We examined the age-related positivity bias and positivity effect using a one-year longitudinal design with a sample that spanned adulthood (N = 374; age range 19-90; M= 47.41; SD= 16.75). Participants answered questions regarding their memories of learning about the outcome of the 2020 U.S. presidential election. Analyses examined the association between age and valence ratings (positive, negative) and ratings of feelings (happy, elated, upset, and shaken) at Time 1, as well as the association with age between change scores for each of those variables, while controlling for who the participant voted for in the election. Results indicate that increased age was associated with reporting feeling less negative at the time of the event, and also remembering feeling more positive (elated and happy) when reconstructing the event one year later, thereby providing evidence of the positivity bias. There was no evidence of an age by valence interaction in a 2 (Valence) x 3 (Age) mixed ANCOVA on the positive and negative change scores, indicating there was not a positivity effect. Depressive symptoms partially mediated the relationship between age and valence variables, indicating that depressive symptoms may be one mechanism for explaining the age-related positivity bias.

